# The addition of genetic testing and cardiovascular magnetic resonance to routine clinical data for stratification of etiology in dilated cardiomyopathy

**DOI:** 10.3389/fcvm.2022.1017119

**Published:** 2022-10-06

**Authors:** Ravi J. Amin, Deborah Morris-Rosendahl, Mat Edwards, Upasana Tayal, Rachel Buchan, Daniel J. Hammersley, Richard E. Jones, Sabiha Gati, Zohya Khalique, Batool Almogheer, Dudley J. Pennell, Arun John Baksi, Antonis Pantazis, James S. Ware, Sanjay K. Prasad, Brian P. Halliday

**Affiliations:** ^1^Cardiovascular Magnetic Resonance Unit, Royal Brompton Hospital, Guy's and St. Thomas' NHS Foundation Trust, London, United Kingdom; ^2^National Heart Lung Institute, Imperial College, London, United Kingdom; ^3^Clinical Genetics and Genomics Laboratory, Royal Brompton Hospital, Guy's and St. Thomas' NHS Foundation Trust, London, United Kingdom; ^4^Department of Inherited Cardiovascular Conditions, Royal Brompton and Harefield Hospitals, Guy's and St. Thomas' NHS Foundation Trust, London, United Kingdom; ^5^MRC London Institute of Medical Sciences, Imperial College London, London, United Kingdom

**Keywords:** dilated cardiomyopathy, genetic testing, cardiac magnetic resonance imaging, etiology, precision stratification

## Abstract

**Background:**

Guidelines recommend genetic testing and cardiovascular magnetic resonance (CMR) for the investigation of dilated cardiomyopathy (DCM). However, the incremental value is unclear. We assessed the impact of these investigations in determining etiology.

**Methods:**

Sixty consecutive patients referred with DCM and recruited to our hospital biobank were selected. Six independent experts determined the etiology of each phenotype in a step-wise manner based on (1) routine clinical data, (2) clinical and genetic data and (3) clinical, genetic and CMR data. They indicated their confidence (1-3) in the classification and any changes to management at each step.

**Results:**

Six physicians adjudicated 60 cases. The addition of genetics and CMR resulted in 57 (15.8%) and 26 (7.2%) changes in the classification of etiology, including an increased number of genetic diagnoses and a reduction in idiopathic diagnoses. Diagnostic confidence improved at each step (*p* < 0.0005). The number of diagnoses made with low confidence reduced from 105 (29.2%) with routine clinical data to 71 (19.7%) following the addition of genetics and 37 (10.3%) with the addition of CMR. The addition of genetics and CMR led to 101 (28.1%) and 112 (31.1%) proposed changes to management, respectively. Interobserver variability showed moderate agreement with clinical data (κ = 0.44) which improved following the addition of genetics (κ = 0.65) and CMR (κ = 0.68).

**Conclusion:**

We demonstrate that genetics and CMR, frequently changed the classification of etiology in DCM, improved confidence and interobserver variability in determining the diagnosis and had an impact on proposed management.

## Introduction

Dilated cardiomyopathy (DCM) is a common phenotype manifest in a diverse group of patients due to a combination of genetic and acquired susceptibility. Treatment includes pharmacological and device therapies for heart failure and uses left ventricular ejection fraction (LVEF) and New York Heart Association symptoms class (NYHA) as the main arbiters (1). The limitations of this approach, which fails to account for the vast heterogeneity in response to therapy and outcome are well recognized (2). More accurate phenotypic stratification may increase consistency, inform treatment and improve outcomes.

Typically, the cause of DCM is determined based on clinical features including exposure to environmental triggers and a family history of cardiomyopathy or sudden death (3). However, exposure to triggers is common amongst the general population and may be poorly defined whenever patients present, having occurred years before symptoms emerge. The amount of alcohol and the length of exposure required for disease expression is unclear and likely to vary between individuals. Even peripartum cardiomyopathy during or following pregnancy, an incredibly well-defined event, can be challenging to diagnose, with doubt arising over the time of onset of symptoms and the pre-existence of an undiagnosed cardiomyopathy. This is likely to impact upon clinician reproducibility in determining the cause of DCM. It is possible that objective data from genetic testing and CMR improves reproducibility and confidence.

Next generation sequencing panels of genes associated with DCM uncover an actionable variant in 20% of cases, rising to 30–40% in the presence of a family history (4). The evidence to support genotype-guided risk stratification is growing (5) and targeted therapies for specific genotypes are on the horizon (6, 7). Recommendations on the use of genetic testing for DCM differ between guidelines, likely due to a lack of evidence from which to draw consensus (1, 8–10). The ACC/AHA/HFSA guidelines recommend targeted testing in patients with suspected inherited etiologies based on family history (1). The ESC guidelines suggest routine use of genetic testing in all patients with DCM (9). It is unclear which approach is more effective. Access is often limited to specialist centers where a small proportion of patients are managed. Research showing an incremental benefit with genetic testing in DCM would support more widespread use and help justify the costs of expanding the availability of genetic testing.

Tissue characterization using CMR also improves phenotypic characterization by assessing the presence, quantity and pattern of myocardial fibrosis (11). As well as improving risk stratification for sudden cardiac death, the pattern of fibrosis and the presence of fibrofatty replacement may implicate certain genotypes or a prior inflammatory insult (12). However, it is not currently mandated for all patients within guidelines (1, 9).

We investigated the incremental value of genetic testing and CMR at the point of diagnosis amongst patients referred for assessment of DCM.

## Methods

We investigated 60 consecutive patients who were referred for diagnostic assessment of DCM and prospectively recruited to our hospital biobank from January to December 2014. At enrolment, all patients underwent genetic testing and cardiovascular magnetic resonance in addition to standard clinical evaluation detailed below. All patients provided informed consent. The study was approved by the National Research Ethics Committee and complies with the declaration of Helsinki.

**Figure 1:**
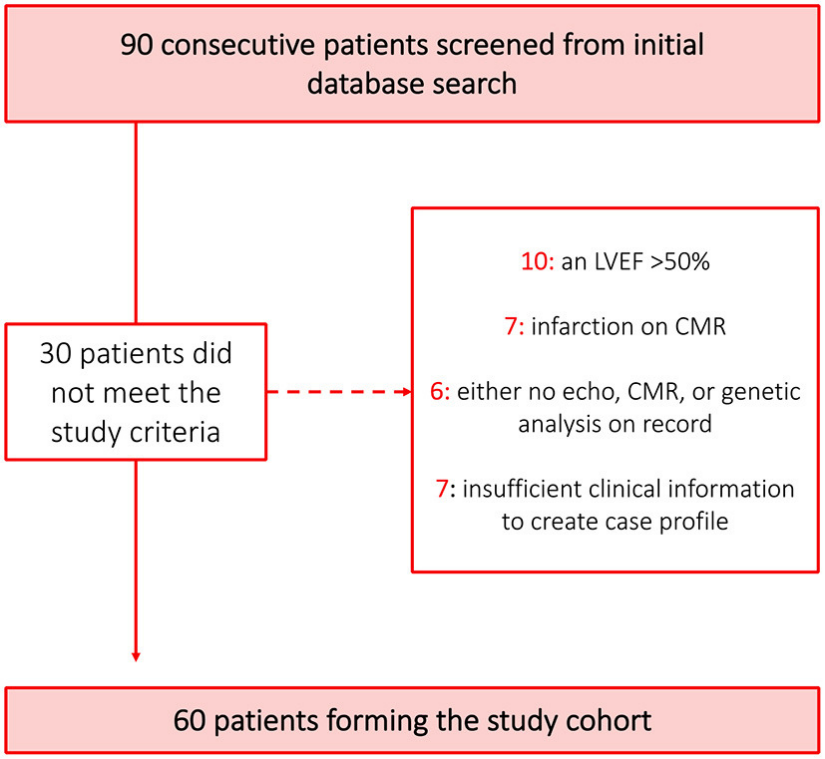
Derivation of cohort. A graphic highlighting the process used to derive the patient cohort used in the study. The 17 patients that were not screened for eligibility were only excluded because the 60 patient threshold desired for the patient cohort had already been met. Clinical information was deemed insufficient where more than one section of the basic investigations could not be completed such as no history of presenting complaint and no blood results.

Inclusion criteria were LVEF ≤ 50% and left ventricular dilatation as defined by age and sex-specific published reference values (13). Exclusion criteria included abnormal loading conditions, such as poorly controlled hypertension or aortic stenosis, ischaemic heart disease (IHD), evidence of acute myocarditis, hypertrophic cardiomyopathy, arrhythmogenic right ventricular cardiomyopathy (ARVC), significant primary valve disease and infiltrative disease. IHD was defined as the presence of ≥50% stenosis in a major coronary artery on coronary angiography, inducible ischaemia on functional testing or infarct patterns of LGE. Each diagnosis of DCM was confirmed by an expert operator trained in CMR and cardiomyopathy. Sixty consecutive patients who met all inclusion criteria and none of the exclusion criteria were identified (Figure 1). None of the patients had a cardiac implanted electronic device at the point of enrolment.

A case profile was created for each patient including the history of presenting complaint, past medical history, 3-generation family pedigree including details of previous clinical screening, key exposures (current and previous medication including chemotherapy, pregnancy history, alcohol exposure, recreational drug use and exercise history), blood test results including full blood count, renal, liver and thyroid function tests, creatine kinase and iron studies, 12-lead electrocardiogram, transthoracic echocardiography and coronary angiography/ischaemia testing. Separate to this, reports of genetic testing and CMR imaging (details below) were collated. CMR images were also available to review. Each case was presented to six cardiologists experienced in the management of cardiomyopathy, in the following order: Part 1) Case history, exposures, and basic investigations (blood test results, electrocardiogram, echocardiography and angiography results), Part 2) addition of genetic results and Part 3) addition of CMR data (Appendix Figure 1). The cardiologist determined the cause of the phenotype at each stage and indicated their confidence in this classification (ordinal score 1–3; 1- low level of confidence, 2- intermediate level of confidence, 3 – high level of confidence) as well as any change to management they would make following the addition of extra data. The classification proposed in the ESC Position Statement, ‘Proposal for a revised definition of dilated cardiomyopathy, hypokinetic non-dilated cardiomyopathy, and its implications for clinical practice' was used (Appendix; Figure 1) (3). Each cardiologist therefore provided three diagnoses, three confidence scores and indicated any changes to management on two occasions (following addition of genetic data and then again following the addition of CMR data) for each case. Six cardiologists adjudicated 60 cases in three steps, providing 1,080 diagnoses.

### Genetic testing

Genetic testing was performed using the TruSight Cardio Sequencing kit (Illumina, San Diego, CA, USA). Testing focused on genes with the most robust evidence in DCM including *TTN, LMNA, MYH7, DSP, TNNT2, TCAP, SCN5A, BAG3, TNNC1, VCL, TPM1, NEXN, RBM20 and PLN* (14, 15). Rare protein-altering variants were identified and interpreted using the ACMG guidelines (16) by the Clinical Genetics and Genomics Laboratory at our center, a regional genetic referral center. For *TTN*, only truncating variants that impact exons constitutively expressed in the heart were included. Variants that were classified as of uncertain significance, likely pathogenic or pathogenic were presented in the case profiles, along with the supporting evidence.

### Cardiac magnetic resonance imaging

CMR was performed on a 1.5 Tesla system (Sonata/Avanto, Siemens, Erlangen, Germany) using a standardized protocol (17). Late gadolinium imaging was performed 10 min after intravenous injection of 0.1 mmol/kg of gadobutrol (Bayer AG, Berlin, Germany) using an inversion-recovery gradient echo sequence. Images were acquired in long-axis planes and short axis slices (8 mm slice thickness with 2 mm gap) in two-phase encoding directions. Inversion times were optimized to null the myocardium. Ventricular volumes were calculated using CMRtools (Cardiovascular Imaging Solutions, London, UK). The presence of non-ischaemic LGE was determined by two independent operators, with a third providing adjudication if necessary. LGE was considered present if seen in both long- and short-axis planes, in two phase-encoding directions and extending beyond the ventricular insertion areas. Short tau inversion recovery imaging was performed to detect myocardial oedema if clinically indicated (17).

### Statistical analysis

All data analysis was conducted using Statistical Package for Social Sciences (IBM SPSS., Version 26.0, NY). A sample size of 60 patients had more than 80% power to detect a 20% rate of change in diagnosis with the addition of genetic testing and CMR data, assuming that 95% of diagnoses are correct based on the most comprehensive information available, at an alpha of 0.05.

Patient characteristics (*n* = 60) and diagnoses are expressed as frequencies where categorical and medians (interquartile range) where continuous. Patient baseline characteristics in those where additional investigations changed etiology and those where it did not, were compared using Fisher Exact test if categorical and the Mann-Whitney *U-*test if continuous variables. Confidence scores were treated as paired ordinal variables. Normality was examined using the Shapiro-Wilks normality test. Confidence scores were compared between the three time points using the Friedman test and between two individual time-points using the Wilcoxon signed rank test. Fleiss' kappa was used to assess interobserver variability in diagnosis with results expressed as kappa (κ) with 95% confidence intervals. For all statistical tests, p < 0.05 was considered statistically significant.

## Results

The baseline characteristics are summarized in Table 1. The majority of patients were men (*n* = 40; 67%) with NYHA class I or II symptoms (*n* = 48; 80%). The median age was 56.5 years. Four patients (7%) had a confirmed family history of DCM, 10 (17%) had a history of moderately excessive alcohol consumption, defined as > 20 units per week, two (3%) had previously received anthracycline chemotherapy and one had a history of cocaine use.

**Table 1:** Patient cohort baseline characteristics (*N* = 60).

**Demographics**	
**Sex**, ***n*** **(%)**	
Male	42 (70.0)
Female	18 (30.0)
Median age, *n* (IQR)	56.5 (43.8–68.0)
**Clinical characteristics**
**NYHA class**, ***n*** **(%)**	
Class I	23 (38.3)
Class II	25(41.7)
Class III	12 (20.0)
Class IV	0 (0.0)
Diabetes, *n* (%)	6 (10.0)
Hypertension, *n* (%)	19 (31.7)
Hypercholesterolaemia, *n* (%)	19 (31.7)
History arrhythmia, *n* (%)*	32 (53.3)
Atrial fibrillation	14 (23.3)
Non-sustained VT	8 (13.3)
Sustained VT/VF	1 (1.7)
**Exposures**	
Hypothyroidism	4 (6.7)
Cancer, *n* (%)	7 (11.7)
Anthracycline chemotherapy^†^	2 (3.3)
Excess alcohol, *n* (%)^§^	11 (18.3)
Recreational drug use, *n* (%)	1 (1.7)
Pregnant at time of diagnosis, *n* (%)	2 (3.3)
**Smoking status**, ***n*** **(%)**	
Non-smoker	28 (46.7)
Current smoker	7 (11.7)
Ex-smoker	25 (41.7)
**Key investigation findings**	
ECG, *n* (%)	55 (91.7)
Sinus rhythm	47 (85.4)
AF	8 (14.5)
LBBB	4 (7.3)
RBBB	3 (5.5)
LVEF, % ^(CMR)^	39.5 (31.5–47.0)
LVEDVi, mL/mm^2^^‡^^(CMR)^	118.5 (102.0–142.0)
LVMi, g/m^2^ ^¶(*CMR*)^	85 (72.0–111.0)
LVIDd, mm ^(echo)^	59 (55–67)
Presence of non-ischaemic LGE, *n* (%)	22 (36.7)
Presence of isolated septal mid-wall LGE, *n* (%)	7 (11.7)
Presence of ring-like LGE, *n* (%)	13 (21.7)
Presence of sub-epicardial LGE, *n* (%)	4 (6.7)
Presence of myocardial oedema	0 (0)
LP/P Variants (%)	13 (21.7)
*TTN*	9 (15.0)
*LMNA*	2 (3.3)
*DSP*	1 (1.7)
*TNNT2*	1 (1.7)
VUS, *n* (%)	4 (6.7)
*DSP*	1 (1.7)
*MYH7*	2 (3.3)
*NEXN*	1 (1.7)
1^st^ degree relative with DCM, *n* (%)	4 (6.7)
Positive FHx and VUS/LP/P variant found, (%)	2 (3.3)
MYH7 (missense)	1 (1.7)
DSP (missense)	1 (1.7)

A summary of the baseline characteristics of the cohort including demographics, clinical characteristics, key exposures, and key investigations completed. All nominal data is presented as a frequency alongside percentage of the total (n, %) and all continuous data is presented as median (IQR). N = 60. VT, ventricular tachycardia; VF, ventricular fibrillation.

*Additional arrhythmias present included sinus arrhythmia, supraventricular tachycardia, and pulseless electrical activity.

^†^Cardiotoxicity was determined based on previous literature and expert opinion.

^§^Excess alcohol was defined as consumption > 20 units per week.

^‡^N = 58 for this parameter as values for two patients were missing from the Biobank Database.

^¶^N = 57 for this parameter as values for three patients were missing from the Biobank Database.

Thirteen patients had a variant in a DCM gene that was classified as likely pathogenic or pathogenic and four had a variant of uncertain significance (VUS); nine had likely pathogenic truncating variants in *TTN*, two had pathogenic missense variants in *LMNA*, two had variants in *DSP (*one pathogenic truncating variant, one splice donor variant of uncertain significance), *one* a likely pathogenic missense variant in *TNNT2*, two missense variants of uncertain significance in *MYH7*, and another a missense variant of uncertain significance in *NEXN* (Tables 2A,2B for variant details). Only two of 17 patients with a variant had a family history of DCM identified on a three-generation family pedigree (one with a VUS in *MHY7* and another with a pathogenic *DSP* variant). A further two patients (3%) without relevant variants on testing had a family history of DCM in a first or second degree relative at the time of assessment.

**Table 2A:** Curation and ACMG classification of variants identified in the cohort – pathogenic and likely pathogenic variants.

**Gene**	**HGVSc**	**Consequence**	**ExAC Freq**	**ACMG class**	**ACMG rules**
DSP	c.1873C>T	Truncating variant	0	Pathogenic	PVS1, PM2, PS4_supp
LMNA	c.568C>T	Missense variant	0	Pathogenic	PM2, PS4, PP1_mod, PS3, PM5, PP3, PP2
LMNA	c. 1622G>A	Missense	3	Pathogenic	
TNNT2	c.415C>T	Missense variant	1	Likely Pathogenic	PM2, PS4_supporting, PP3
TTN	c.52254G>A	Loss of function variant	0	Likely Pathogenic	PVS1_strong, PM2
TTN	c.44364delC	Loss of function variant	0	Likely pathogenic	PVS1_strong, PM2
TTN	c.3380 + 1G>C	Splice donor variant	0	Likely pathogenic	PVS_1 strong
TTN	c.44281 + 1G>A	Splice donor variant	1	Likely pathogenic	PVS1_strong, PM2
TTN	c.76355G>A	Loss of function variant	0	Likely pathogenic	PVS1_strong, PM2
TTN	c.67567delG	Loss of function variant	0	Likely pathogenic	PVS1_strong, PM2
TTN	c.89216delC	Loss of function variant	0	Likely pathogenic	PVS1_strong, PM2
TTN	c.59226T>G	Loss of function variant	0	Likely pathogenic	PVS1_strong, PM2
TTN	c.42235C>T	Loss of function variant	1	Likely pathogenic	PVS1_strong, PM2

Summary of the pathogenic and likely pathogenic genetic variants identified in the DCM cohort using the TruSight Cardio Sequencing kit (Illumina, San Diego, CA, USA) with variants interpreted using the ACMG guidelines. ACMG, American College of Molecular Genetics; HGVSc, Human Genome Variant Society code; VUS, variant on unknown significance.

**Table 2B:** Curation and ACMG classification of variants identified in the cohort – variants of unknown significance.

**Gene**	**HGVSc**	**Consequence**	**ExAC Freq**	**ACMG Class**	**ACMG Rules**
DSP	c.1574 + 3_1574 + 6delAAGT	Splice donor variant	0	VUS	PP3, PM2
MYH7	c.4435A>G	Missense variant	5	VUS	
MYH7	c.5187G>T	Missense variant	1	VUS	
NEXN	c.586C>T	Missense variant	30	VUS	

Summary of the variants of unknown significance identified in the DCM cohort using the TruSight Cardio Sequencing kit (Illumina, San Diego, CA, USA) with variants interpreted using the ACMG guidelines. ACMG, American College of Molecular Genetics; HGVSc, Human Genome Variant Society code; VUS, variant on unknown significance.

**Figure 2:**
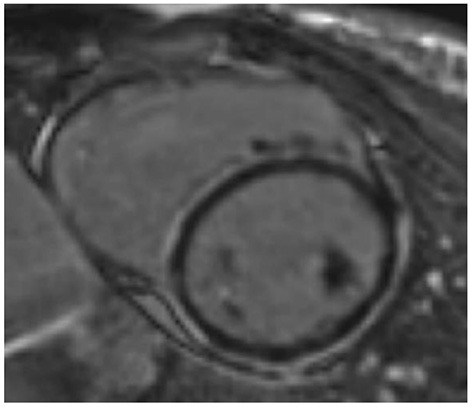
Ring-like pattern of fibrosis on cardiovascular magnetic resonance. A patient with dilated cardiomyopathy secondary to a truncating variant in *DSP*, with ring-like late gadolinium enhancement on CMR.

On CMR, the mean LVEF was 38% (SD 11) and LVEDVi 128 ml/m (2) (SD 37). Overall, 22 (37%) had non-ischaemic fibrosis on late gadolinium enhancement imaging, including 13 (22%) patients with a ring-like pattern of circumferential or near-circumferential mid-wall/sub-epicardial enhancement. Of those with ring-like patterns of fibrosis (Figure 2), three (23%) had a variant identified, two with pathogenic *LMNA* variants and one with a pathogenic *DSP* variant.

### Stratifying etiology

**Figure 3:**
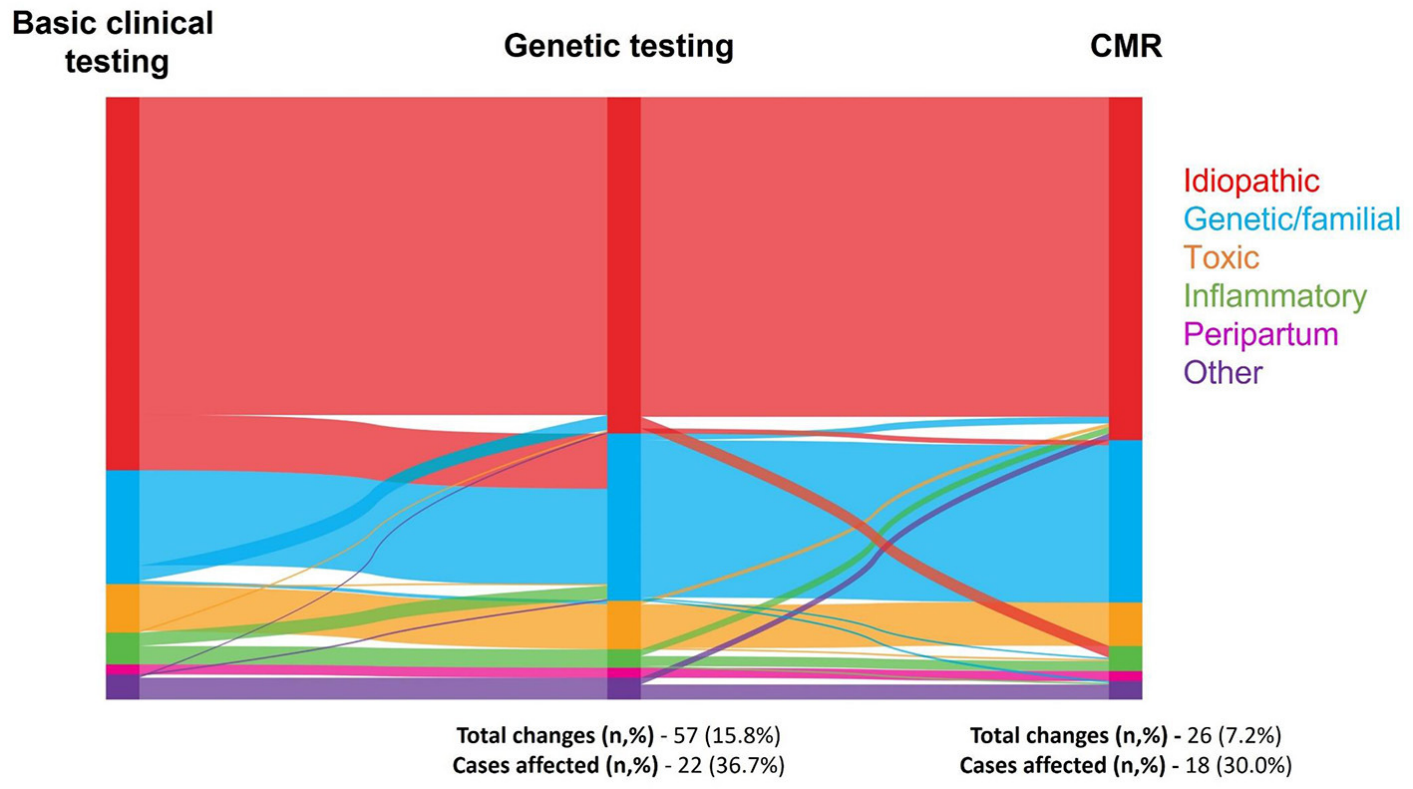
Sankey diagram of change in etiology by investigation. A Sankey diagram illustrating the number of times etiology was changed when genetic testing and subsequently CMR results were added to the information provided by the basic investigations, where *N* = 360 each time an investigation was added. Etiologies of dilated cardiomyopathy were grouped into idiopathic, genetic/familial, toxic (comprised of iron overload, recreational drugs, anti-neoplastic drugs, other medications, and alcohol), inflammatory (comprised of viral myocarditis and autoimmune), peripartum, and other (comprised of other, nutritional deficiency, and hypothyroidism).

Idiopathic DCM was the most common classification adjudicated following the presentation of clinical data, comprising 223 of 360 (62%) diagnoses. This reduced to 201 (56%) after genetic testing and 205 (57%) following the addition of CMR results (Table 3). In parallel, the number of genetic diagnoses increased from 68 (19%) with routine clinical data, to 100 (28%) with genetic data and 97 (27%) with CMR and genetic data. Overall, the sequential addition of genetic testing and CMR prompted changes in diagnosis in 22 (37%) and 18 (30%) of the 60 cases (Figure 3). The addition of genetic testing resulted in changes to 57 of the overall 360 (16%) diagnoses. The majority of these changes (44 of 360, 12%) reflected a new diagnosis of genetic DCM and in each of these cases, a likely pathogenic or pathogenic variant was identified (Figure 3). CMR results, subsequently triggered changes to diagnosis in a further 26 of 360 (7%) instances. Of note, the number of cases of suspected inflammatory cardiomyopathy, either viral myocarditis or autoimmune disease, reduced by eight following the identification of a relevant variant on genetic testing. Conversely the number of cases of inflammatory cardiomyopathy increased by four following CMR due to the identification of tissue characteristics consistent with the diagnosis. The two cases of suspected iron overload were discounted following CMR with T2^*^ measurement.

**Table 3:** Frequency of DCM etiologies by investigation.

**Etiology**	**Basic investigations *n* (%)**	**Genetic testing** ***n* (%)**	**CMR *n* (%)**
Genetic/familial	68 (18.9)	100 (27.8)	97 (26.9)
Alcohol	9 (2.5)	10 (2.8)	10 (2.8)
Iron overload	2 (0.6)	2 (0.6)	0 (0.0)
Recreational drugs	2 (0.6)	2 (0.6)	2 (0.6)
Anti-neoplastic	14 (3.9)	13 (3.6)	12 (3.3)
Other meds*	2 (0.6)	2 (0.6)	2 (0.6)
Hypothyroidism	5 (1.4)	5 (1.4)	5 (1.4)
Idiopathic	223 (61.7)	201 (55.8)	205 (56.9)
Viral Myocarditis	12 (3.3)	8 (2.2)	11 (3.1)
Autoimmune	7 (1.9)	3 (0.8)	4 (1.1)
Peripartum	6 (1.7)	6 (1.7)	6 (1.7)
Nutritional deficiency	1 (0.3)	1 (0.3)	0 (0.0)
Other^†^	9 (2.5)	7 (1.9)	6 (1.7)

The frequency with which each etiology was diagnosed at each of the three time points, when basic investigations were presented, when genetic testing was added, and finally when CMR results were added. N = 360 at each of the three points of measure.

^*^The ‘Other Meds' comprised of infliximab.

^†^The ‘Other' etiologies included four tachy-induced cardiomyopathies, three hypertensive cardiomyopathies (HTN CM), one ischaemic cardiomyopathy (ICM), and one left ventricle non-compaction (LVNC) following basic investigations, three tachy-induced cardiomyopathies, two HTN CM, one ICM, and one LVNC following genetic analysis, and finally, three tachy-induced cardiomyopathies, two HTN CM, and one possible athletic remodeling following CMR.

### Confidence scores

**Figure 4:**
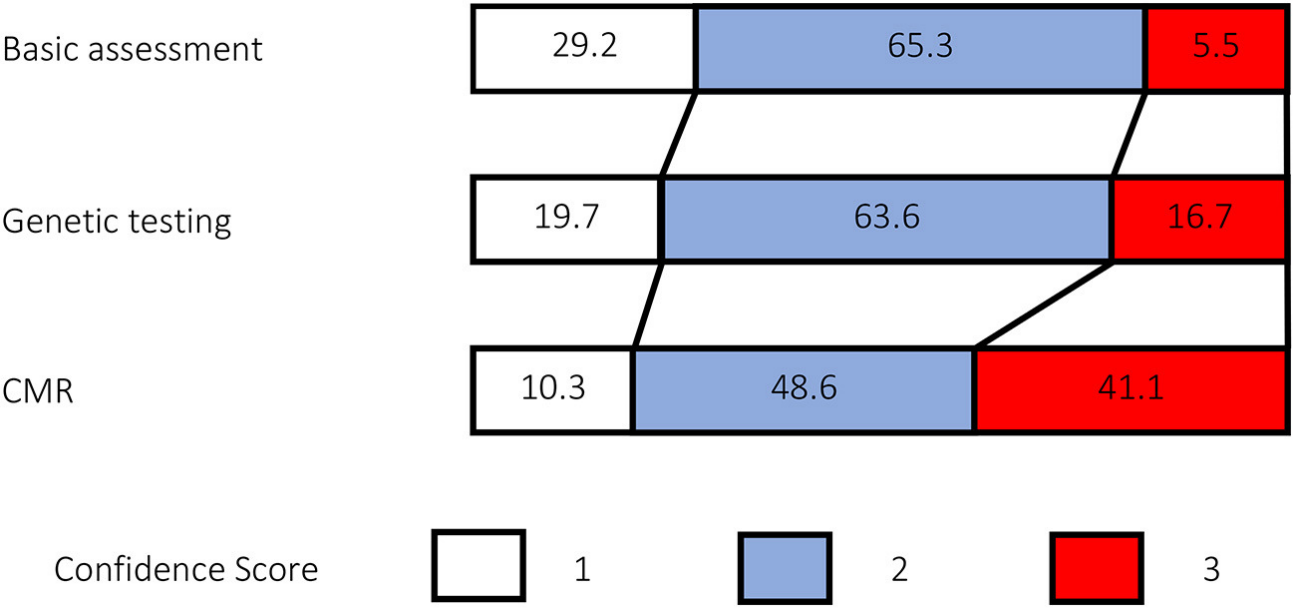
Comparison of confidence scores. Confidence scores were recorded each time the expert was asked to diagnose etiology, resulting in *N* = 360 confidence scores at each set of investigations. Numbers in each color-coded box reflect the percentage frequency of each score.

The addition of genetic testing and CMR findings to routine clinical investigations increased the confidence of clinicians in stratifying the cause of the DCM (Figure 4). Comparisons showed a significant difference between the groups overall (*p* < 0.0005) and also between individual groups at each step in the process (genetic and clinical data vs clinical data, *p* < 0.0005; CMR, genetic and clinical data vs. genetic and clinical data, *p* < 0.0005) The number of diagnoses made with low confidence reduced from 105 (29.2%) with routine clinical data to 71 (19.7%) following the addition of genetics and 37 (10.3%) with the addition of CMR. The number of diagnoses made with high confidence increased from 20 (5.5 %) with routine clinical data to 60 (16.7%) following the addition of genetics and 150 (41.1%) with the addition of CMR.

### Interobserver variability in determining etiology

Fleiss's kappa demonstrated moderate agreement in diagnoses following basic investigations (κ = 0.44 [95% CI 0.42–0.46]), and good agreement following the addition of genetic testing (κ=0.65 [95% CI 0.63–0.67]), and, later, CMR (κ = 0.68 [95% CI 0.66–0.70]).

### Change in management

Genetic testing results prompted 101 (28%) changes in management, with 84 (23%) of those management changes relating to the recommendation to carry out clinical family screening and 4 (1%) relating to a change to device therapy (Table 4). Instances where devices therapy was recommended following genetic testing included cases where a pathogenic *LMNA* variant was identified and another where a pathogenic *DSP* variant was identified. The addition of CMR results triggered 77 (21%) changes in management with 21 (6%) involving family screening and 21 (6%) involving the new recommendation of device therapy based on the presence of mid-wall fibrosis. Medical therapy was intensified on 15 occasions (4%) based on the perception of a higher risk phenotype after the genetic or CMR results. Lifestyle advice, including advice on alcohol and exercise was changed on eight occasions (2%).

**Table 4:** Frequency of change in management by investigation.

**Investigation** **added**	**Total changes *n* (%)**	**Cases affected** ***n* (%)**	**Breakdown by type of management changed**	
Genetic testing	101 (28.1%)	54 (90.0%)	Medical therapy:	3
			Device therapy:	4
			Lifestyle therapy:	4
			Family screening:	84
			Other:	2
CMR	77 (21.4%)	50 (83.3%)	Medical therapy:	12
			Device therapy:	21
			Lifestyle therapy:	4
			Family screening:	21
			Other:	15

The number of times management was changed following the addition of genetic testing and subsequently CMR results to the information provided by the basic investigations. N = 360 for each investigation added. Frequencies are reported with a percentage compared to N and are expressed as n (%). For the ‘Cases Affected' column, N = 60 cases. The changes to management divided into categories in the fourth column on the left where frequencies reported as n (%).

## Discussion

Overall, our data demonstrate that the addition of genetic testing and CMR to routine clinical investigations resulted in frequent changes to diagnosis in patients with DCM. This improved diagnostic confidence as well as interobserver agreement in a clinical setting with subsequent impact on management. Taken together, the findings suggest that the regular and widespread implementation of genetic testing and CMR, as recommended in the latest 2021 ESC Heart Failure Guidelines may increase the reproducibility and robustness of our approach to diagnosing and managing DCM (9).

The improvement in diagnostic confidence and interobserver variability highlights the more objective nature of expertly interpreted genetic testing and CMR when performed using established guidelines and frameworks. This is compared to the more variable and subjective process of taking and interpreting a clinical history, which frequently includes poorly defined events. Based on available data, the characteristics of our cohort appear to reflect that of a typical, consecutively recruited DCM population (17, 18). The number of cases with a likely pathogenic or pathogenic variant aligns with previous data, including the predominance of truncating variants in *TTN* (19, 20). As expected, the addition of genetic testing triggered changes in diagnosis through an increase in the diagnosis of genetic disease. Testing resulted in a 10% increase in the number of genetic diagnoses. Many of the patients with new diagnoses of genetic cardiomyopathies were suspected to have idiopathic disease or inflammatory forms of cardiomyopathy based on initial investigations. This has important implications for family members who may not even have been offered clinical screening following the diagnosis of an inflammatory cardiomyopathy in the index case and may now be offered predictive genetic testing to establish future risk. Genetic variants were frequently identified in probands without a preceding family history at presentation. This confirms the importance of clinical screening of 1st degree family members and the need to consider genetic testing even in the absence of a clear familial phenotype. It is therefore likely that a targeted approach to genetic testing, as recommended in the latest ACC/AHA/HFSA guidelines (21) will fail identify many patients with a monogenic cause of DCM and that a more widespread adoption to testing, provided this is adequately resourced, is preferable. Genetic DCM is characterized by incomplete penetrance and variable expressivity, particularly with truncating variants in TTN. The likelihood of expressing a phenotype and the severity of expression is likely related to gene-environmental interactions as well as polygenic risk (22, 23). Gene positive family members without a phenotype may carry risk of developing the disease and it is possible that this may be better established through the use of cascade genetic testing within a multidisciplinary setting. This not only helps determine follow-up strategy but also guides lifestyle advice and monitoring around pregnancy and other haemodynamic stressors. In one patient without a preceding family history, a *de novo* pathogenic missense variant in *LMNA* was identified. This prompted screening of at-risk offspring and guided decision making about risk stratification and device implantation. This confirms the importance of maintaining an open mind regarding the possibility of a genetic cause in the absence of a clear family history. Though current genotype-guided management changes largely center around family screening and device implantation, with an increasing body of gene-specific outcome data and the emergence of targeted molecular therapies, a growing role in guiding therapy is inevitable.

Though fewer in number, the addition of CMR results triggered further changes to diagnoses. This included new diagnoses of inflammatory disease based on typical tissue characteristics and the exclusion of myocardial iron overload following T2^*^ imaging (12, 24). The use of T2^*^ imaging to guide therapy and improve outcomes in patients with myocardial iron overload is well established (25). The management of DCM due to chronic inflammation is still debated. However, it is possible that CMR-based tissue characterization may guide the use of targeted anti-inflammatories. Whilst certain patterns of LGE may favor a genetic cause, the overall number of genetic diagnoses fell slightly after CMR. Each case which changed from a suspected genetic cause following CMR was genotype negative and the adjudicator felt that the overall balance of clinical suspicion changed in favor of an alternative diagnosis rather than a genetic cause. Conversely, some adjudicators recommended the introduction of clinical screening of first-degree family relatives after CMR. This was often in cases where an alternative acquired etiology was initially suspected but subsequently not supported by CMR. This prompted adjudicators to consider further investigation in unexplained gene elusive disease. It is also possible that certain patterns of fibrosis or fibrofatty infiltration led clinicians to consider the possibility of genetic disease as an alternative to other suspected etiologies, albeit perhaps less likely overall after the absence of actionable genetic findings and a family history. Interestingly, around a quarter of patients with ring-like LGE on CMR had a variant in *LMNA* or *DSP*. Both genotypes are associated with adverse arrhythmic outcomes (5). This is likely to, at least partly, explain the high-risk nature of ring-like LGE. It should be noted that the gene panel used for the study did not include *FLNC and DES*. This is a limitation, however given the prevalence of such variants in other representative cohorts (26), we believe that the overall results of the study would have remained similar if these genes were included in the panel. It is also possible that some inflammatory phenotypes may produce near circumferential patterns of LGE. An overlap between genetic and inflammatory cardiomyopathies is also increasingly recognized (27). The well documented association between mid-wall fibrosis and ventricular arrhythmia (17) was the main driver of proposed changes to device therapy following CMR. As we await randomized trials examining the use of mid-wall fibrosis to select patients for implantable cardioverter defibrillators, this is currently used to predominantly guide borderline cases.

### Limitations

This study was a small, single-center study. Whilst such studies are susceptible to selection bias, the baseline characteristics of the cohort are similar to those of other consecutively recruited cohorts of DCM (18, 28). This suggests that the current cohort was representative of the wider population. The frequency and type of genetic variants are also very much in-keeping with previously published data (18, 28).

Due to the order of tests, this study did not examine the incremental value of CMR in addition to routine clinical data. The primary focus of this manuscript was to assess the impact of genetic testing, which represents the more prominent gap in the evidence. Furthermore, whilst the absence of a consensus diagnosis for baseline etiology may have generated additional variability, we decided to keep the responses of each adjudicator independent rather than reaching an initial consensus diagnosis to better allow for assessment of interobserver variability, a key secondary objective.

It is also possible that the frequency of inflammatory DCM diagnoses may have been underestimated in this paper as patients only underwent T2-weighted imaging if the reviewing CMR physician felt this to be clinically indicated.

## Conclusion

This study demonstrates the incremental value of genetic testing and CMR in the stratification of DCM, leading to greater reproducibility and improved diagnostic confidence. These investigations also prompted a change in clinical management in a small number of patients. With increasing amounts of genetic and CMR-based outcome data and emerging targeted molecular therapies, such investigations will play central roles in guiding precision management based on more comprehensive understanding of the disease. Widespread adoption of genetic testing for all cases of DCM should be an important ambition.

## Data availability statement

The data analyzed in this study is subject to the following licenses/restrictions: Pseudonymised data can be made available for suitable projects where patient consent allows. Requests to access these datasets should be directed to the corresponding author.

## Ethics statement

The studies involving human participants were reviewed and approved by South Central - Hampshire B REC and our Insitutional Review Board. The patients/participants provided their written informed consent to participate in this study.

## Author contributions

BH and RA designed the study. RA, DM-R, ME, UT, RB, DH, RJ, SG, ZK, BA, DP, AB, AP, SP, and BH participated in data acquisition and analysis and interpretation. RA drafted the manuscript. All authors contributed to the article and approved the submitted version.

## Funding

The study was supported by the NIHR Royal Brompton Cardiovascular Biomedical Research Unit. BH is supported by a BHF Intermediate Fellowship (FS/ICRF/21/26019), Rosetrees Trust and a Clinical Lecturer Starter Grant from the Academy of Medical Sciences (SGL021/1025). JW is supported by the British Heart Foundation [RE/18/4/34215 and SP/17/11/32885], NIHR Imperial College Biomedical Research Center, Sir Jules Thorn Charitable Trust [21JTA]. BH, JW, and SP are supported by the NHLI Foundation and Royston Centre for Cardiomyopathy Research.

## Conflict of interest

Author DP has received research funding from Siemens. Author JW has consulted for Myokardia (now BMS), Pfizer and Foresite Labs. The remaining authors declare that the research was conducted in the absence of any commercial or financial relationships that could be construed as a potential conflict of interest.

## Publisher's note

All claims expressed in this article are solely those of the authors and do not necessarily represent those of their affiliated organizations, or those of the publisher, the editors and the reviewers. Any product that may be evaluated in this article, or claim that may be made by its manufacturer, is not guaranteed or endorsed by the publisher.
