# Supplementary material for: The addition of genetic testing and cardiovascular magnetic resonance to routine clinical data for stratification of etiology in dilated cardiomyopathy
Source: Front Cardiovasc Med. 2022 Oct 6;9:1017119. doi: 10.3389/fcvm.2022.1017119 (PMC9582287; doi:10.3389/fcvm.2022.1017119)
Supplement: Supplementary file 1 [file Data_Sheet_1.docx]

**Supplementary appendix**

**Appendix figure 1: Sample Patient Case and Data Collection Tool**

Patient Case Format


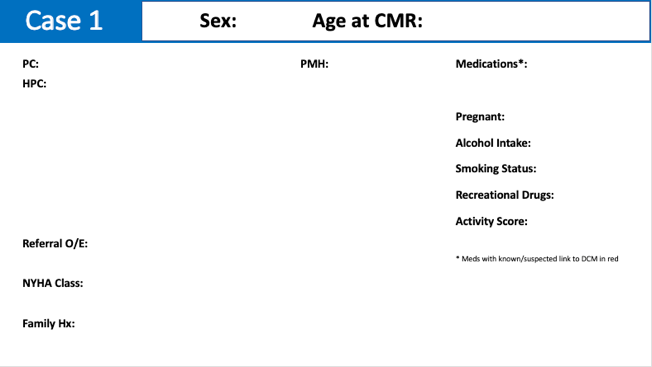

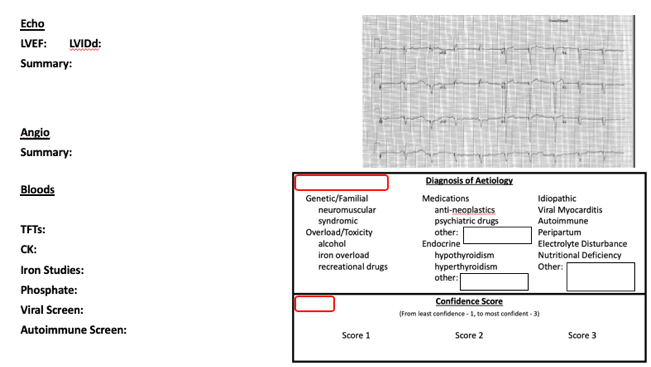

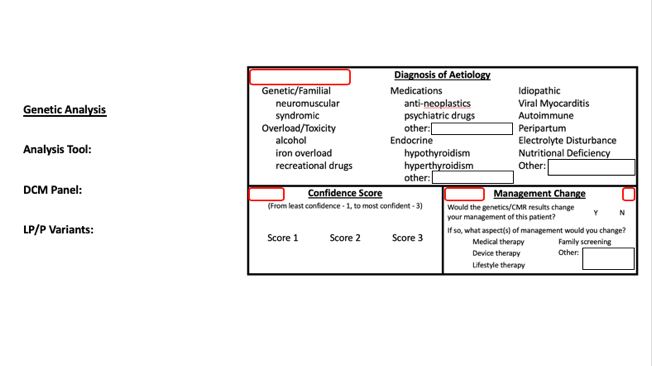

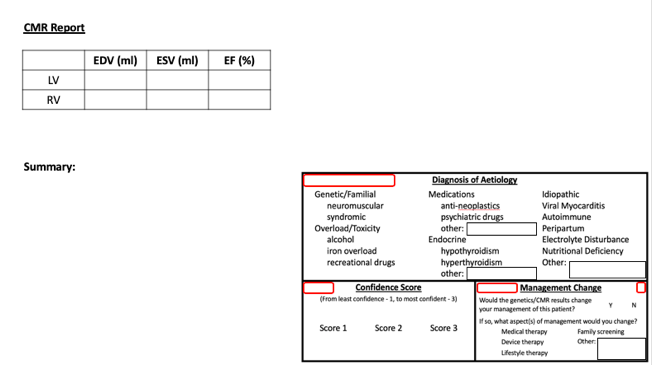


Sample Data Collection Tool


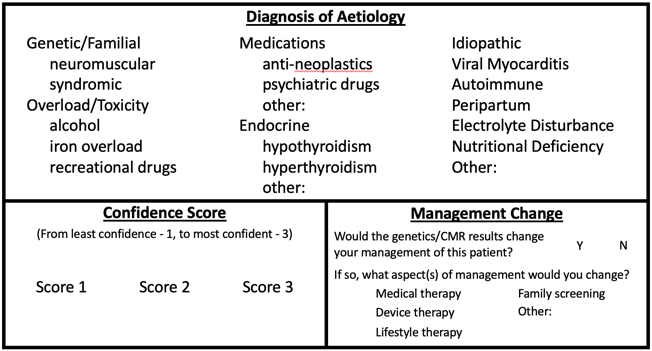

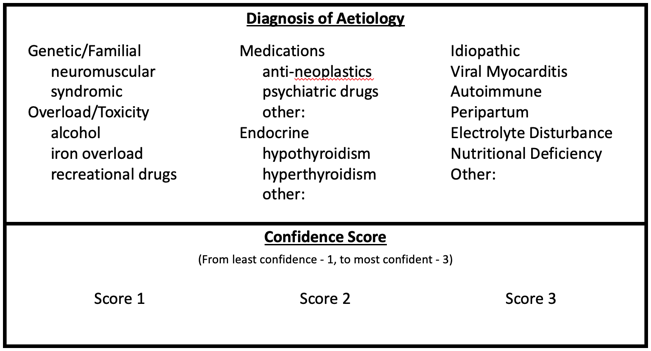


Figure 1: Each of the 60 patients had a case profile constructed which was broken up into 3 parts: (1) clinical history and basic investigations, (2) genetic testing results, and (3) CMR results. Data was collected at the end of each part using the data collection tools (DCT) on each slide. The last two panels show enlarged versions of the collection tool. The DCT on the bottom left is used after part 1 and the DCT on the bottom right used after parts 2 and 3.

**Appendix table 1:**

| **Table 1: Demographics, Clinical Characteristics, and Exposures by Presence of Change in etiology following Addition of Genetic Testing and CMR Results** | | | |
| --- | --- | --- | --- |
|  | No Change in etiology (N=24) | Change in etiology (N=36) | p-value |
| Median Age, n(IQR) | 63 (46.25-68.75) | 55 (43 – 67.5) | .32 |
| Sex, n(%)  Male  Female | 18 (75.0)  6 (25.0) | 24 (66.7)  12 (33.3) | .57 |
| NYHA Class, n(%)  Class I  Class II  Class III  Class IV | 11 (45.8)  9 (37.5)  4 (16.7)  0 (0.0) | 12 (33.3)  16 (44.4)  8 (22.2)  0 (0.0) | .66 |
| Diabetes mellitus, n(%) | 3 (12.5) | 3 (8.3) | .68 |
| Hypertension, n(%) | 10 (41.6) | 9 (25.0) | .26 |
| Hypercholesterolaemia, n(%) | 11 (45.8) | 8 (22.2) | .09 |
| History of Arrhythmia, n(%)*  Atrial Fibrillation  Non-sustained VT  Sustained VT/VF | 15 (62.5)  7 (29.1)  4 (16.7)  0 (0.0) | 17 (47.2)  7 (19.4)  4 (11.1)  1 (11.1) | .42  .54  .70  1 |
| Family History, n(%) | 3 (12.5) | 5 (13.9) | 1 |
| Activity Score  1  2  3  4 | 2 (8.3)  15 (62.5)  7 (29.1)  0 (0.0) | 9 (25.0)  22 (61.1)  5 (13.9)  0 (0.0) | .15 |
| Hypothyroidism, n(%) | 2 (8.3) | 2 (5.6) | 1 |
| Cancer, n(%)  Anthracycline Chemotherapy^†^ | 2 (8.3)  1 (4.2) | 5 (13.9)  1 (2.8) | .691  1 |
| Pregnant at time of diagnosis, n(%) | 1 (4.2) | 1 (2.8) | 1 |
| Excess Alcohol, n(%)^§^ | 4 (16.7) | 7 (19.4) | 1 |
| Recreational Drug Use, n(%) | 1 (4.2) | 0 (0.0) | .40 |
| Smoking Status, n(%)  Non-Smoker  Current Smoker  Ex-smoker | 12 (50.0)  2 (8.3)  10 (41.7) | 16 (44.4)  5 (13.9)  15 (41.7) | .87 |

A comparison of the key clinical characteristics in cases where the addition of genetic testing and CMR results changed or did not change aetiological diagnosis. All nominal data is presented as a frequency alongside percentage of the total (n, %) for each group and all continuous data is presented as median (IQR). Comparisons using Mann Whitney U test for continuous data and Fisher’s Exact test for categorical data. N = 24 for cases with No Change in etiology and N = 36 for cases with a Change in etiology.

VT = ventricular tachycardia, VF = ventricular fibrillation

*Additional arrhythmias encountered include sinus arrhythmias, supraventricular tachycardias, and pulseless electrical activity

† Cardiotoxicity was determined based on previous literature and expert opinion

§ Excess alcohol was defined as consumption > 20 units per week
